# The Sexual Life of the Child

**Published:** 1913-03

**Authors:** 


**The Sexual Life of the Child.** By Dr. ALBERT MOLL. Translated from the German by Dr. Eden Paul. Pp. ix, 339. London: George Allen & Company Ltd. 1912. 15s. net.

It was inevitable that sexual life in the child both normal and morbid should receive investigation, and Dr. Moll's qualifications for the task are undoubted. Hitherto in England, with one eminent exception, the subject of sexual life and its perversions has been considered with much reserve. It may be that, adopting the attitude of Mr. Podsnap, we have indignantly put the question behind us, as un-English, or perhaps that we have been, although far from unenlightened, after the manner of Albion, cloaked with a perfidious modesty. It must be admitted that science should have no scruple in unveiling these secret and discreditable perversions for study; it is only to be stipulated that they be not unduly paraded with all their repugnant features, but inspected in chasteness of spirit and revealed within carefully circumscribed limits. We hold it, above all, both possible and needful to deal with the whole subject tersely and with ultra-scientific dryness, avoiding all superfluous clinical details, being hypercritical in the elimination of anything that can appeal to a prurient imagination. The supposed limitation in issue of a book to certain professions is not so protective against abuse as the punctilious handling of the subject-matter. We have here a closely-printed work of over three hundred pages, and though, on the whole, it is not irrelevantly charged with offensive features, we consider it unnecessarily prolix and indulgent in detail, like most books of its kind. The author drops a warning during its course as to the need for reserve in the acceptance of a sexual interpretation of certain conduct in the young, but the general effect of the work is to arouse a distasteful suspicion of the child's sexual neutrality, and to undermine our reasonable conceptions as to the general and virtual purity of child-life. We should confess to a sense of shock did we wholly accept Moll's statements as to the large proportion in which there exists a latent sexuality in many of the games, the habits, the natural or erratic friendships of the young, as also his pronouncement in regard to the frequent and gross sexual proclivities of children, whether innocently acquired or the outcome of innate vice. Unless, pharisaically, we gaze at this deplorable aspect of the child's sexual life as one without our own shores, we should in adopting Moll's views allow an attitude in ourselves of unpleasant inquisitiveness into behaviour in the young, hitherto disregarded; a quite unpalatable state, and one we trust by no means often necessary. A fresh and consuming anxiety, too, would be afforded to those clergymen, lawyers and schoolmasters who are supposed to share with medical men the interests of child-study.

We find in this book details of every vice and perversion concerned with sexual life in the child; their auto- or hetero-genesis, their indictment or apology, prevention and treatment. From the jurisprudential point of view, Moll utters the warning that the acceptance of the child-witness as one inexperienced and unprejudiced in sex-matters is a grave error. The possible strength and variety of sexual imagination must be allowed for. Another item, redness of the genitalia in female children is not to be assumed as necessarily the result of a criminal assault. We note that Moll does not endorse Freud's views as to the sexual origin of neuroses, nor the reliability of his psycho-analytic method. There is lengthy discussion, worthy of careful study, as to the desirability, time and method of enlightenment of the young in sexual matters. Finally, those who desire to obtain at all costs a both comprehensive and detailed account of every aspect of the child's sexual life will not search this book in vain. To such we commend it. Dr. Eden Paul, the translator, has done his task most efficiently.

